# Supplementary material for: Rapid turnover of life-cycle-related genes in the brown algae
Source: Genome Biol. 2019 Feb 14;20:35. doi: 10.1186/s13059-019-1630-6 (PMC6374913; doi:10.1186/s13059-019-1630-6)
Supplement: Supplementary file 3 — Protein alignments, corresponding Gblocks html file and CDS sequences for the lineage conserved gametophyte-biased genes. (ZIP 2109 kb) [file 13059_2019_1630_MOESM3_ESM.zip › Con_GA_alignments/Ec-14_003580.aa_ali.fasta-gb.htm]

Ec-14\_003580.aa.fasta.trans.aa\_ali.fasta


## Gblocks 0.91b Results

Processed file: **Ec-14\_003580.aa.fasta.trans.aa\_ali.fasta**  
Number of sequences: **2**  
Alignment assumed to be: **Protein**  
New number of positions: **319** (selected positions are underlined in blue)

```
                         10        20        30        40        50        60
                 =========+=========+=========+=========+=========+=========+
Ec-14_003580     MESMANGCRDSADNCETKSFELSKTNKNVPASPAAGTASPPRHALLAPSFSIVEDTKG--
MSTRG.34604.1    -----------HHHCDHRDHSQGFFRQEKA--GAVCTASRDSSPRMAPSFSVLQDTEDGD
                                                  #######################    


                         70        80        90       100       110       120
                 =========+=========+=========+=========+=========+=========+
Ec-14_003580     -------STDVTPPPDIPPSEGDPWGDGGSPLTARSASSLGRLVPERTARAEEEAARGPP
MSTRG.34604.1    QPRALSPPVELDVPPEIPLPWGSPGDIVSSSSTSSSSSFPGRPVPERTAMYVSQQKSFHA
                              ####################################           


                        130       140       150       160       170       180
                 =========+=========+=========+=========+=========+=========+
Ec-14_003580     ------ADRSSHWAVAAGPNKAQRETEGTPPTG---------------------------
MSTRG.34604.1    NISDGFIDLSSPAGGVHGQGGGGRGRGGRAPVGEGEKDDQSCVITVEAGTITSRKGAICG
                        ##########################                           


                        190       200       210       220       230       240
                 =========+=========+=========+=========+=========+=========+
Ec-14_003580     SLSLTPSEVVWRSSSSDCGRKGGCEEEYVGRTERRTRASSNPVPIGGDLGSSGQTKGTGG
MSTRG.34604.1    SSSAPTVDDTRSATSSTCRGTQAPHEKQLEQIAPPIVSAVRPSPKTTWESKSGCGRQDGG
                               ############               ###################


                        250       260       270       280       290       300
                 =========+=========+=========+=========+=========+=========+
Ec-14_003580     QE---QKLDELSLPTRWTPIEACSLAAEDEKARSDQNERRHERG-------ATPPLSQVH
MSTRG.34604.1    QRDITGPAQEVEASPSAVPVGEPSLSASMHSVNVHAVSSKKSEGDTRLVLEEAKELEQAA
                 #        ##################                                 


                        310       320       330       340       350       360
                 =========+=========+=========+=========+=========+=========+
Ec-14_003580     SESVLRGSPREPTGNLEGDGGAGG-DLMLFRTDVSRHLVSGEQPALFFLRPSENLSVTRI
MSTRG.34604.1    TRLLSHVQSKESTRNEKGDSYMPREDLVHFRTDVSRHLVSGERPALFFLRPNESLAVTRI
                                          ###################################


                        370       380       390       400       410       420
                 =========+=========+=========+=========+=========+=========+
Ec-14_003580     EGTGRLLVTLREEAGSAAAVVIDGATVHDSAAHFGHEAVQTDHRFPYVAGHRYEFQVAPA
MSTRG.34604.1    DGSGRLLVTLREEAG-AEIVAVDGAIVHDSAAHFGDAAINTGHRFPYVAGHRYEFRVVPA
                 ############### ############################################


                        430       440       450       460       470       480
                 =========+=========+=========+=========+=========+=========+
Ec-14_003580     QGVELAVRGGGGGESRQGAEASQGCAANTSSGLHVVFSVMSAYSKGARVCQKDAMRLSLR
MSTRG.34604.1    QGVELAVRGNGNGQGK-AAPCAPQAKKTPITGLNVSFSVTSAYSTGARLSERDAMQVALP
                 #############                  #############################


                        490       500       510       520       530       540
                 =========+=========+=========+=========+=========+=========+
Ec-14_003580     TFEAPAANGSRQP--TKPKSPATAMTKDPPTPSNLDASATATVDEGISAPRRPSLKEQDH
MSTRG.34604.1    TFEATPPTDLAQHPATLVSSAPTAARKTQPSPCFVAATDEARLSP------NARFQGPDS
                 ############   ##########################                   


                        550       560       570       580       590       600
                 =========+=========+=========+=========+=========+=========+
Ec-14_003580     AGDQRRTPSPSVAAALLASCLSPPPEVKHNHLIGTAPREEPSAPTAGVQPPAAAPKTPQP
MSTRG.34604.1    VGDQR-SSQVTTEEALVGPCVSRPS-----------------------------------
                               ##########                                    


                        610       620       630       640       650       660
                 =========+=========+=========+=========+=========+=========+
Ec-14_003580     SSSTRTAAAAAAEGGGVQGGVRGRPVQSAETTGGNQSAGAATRGQRQPGSGVDPASTPYV
MSTRG.34604.1    ----------------VGGGPLATTLSVVEASA------------RRPG---DSAPVPI-
                                                                             


                        670       680       690       700       710       720
                 =========+=========+=========+=========+=========+=========+
Ec-14_003580     TVSGRPPPSGMEGAIYPSAERLRWNPAWEQQRLLPQSPSAPAEMLPPSHAPSLPHIRRHV
MSTRG.34604.1    -------------------------------------PT---------------------
                                                                             


                        730       740       750       760       770       780
                 =========+=========+=========+=========+=========+=========+
Ec-14_003580     DSSSSSASPRSTLGRLEPSGVAGSHHVPRDPAIGAPHRYVLPPGYPAGEHPVQSKRYVGA
MSTRG.34604.1    ------------------------------------------------------------
                                                                             


                        790       800       810       820       830       840
                 =========+=========+=========+=========+=========+=========+
Ec-14_003580     EDAPGTKASRHYYQPAHEERHHGAPRYHYHRYVSDGQRDVASPGHQAGEGVRELEWNRGR
MSTRG.34604.1    ------------------------------------------------------------
                                                                             


                        850       860       870       880       890       900
                 =========+=========+=========+=========+=========+=========+
Ec-14_003580     RKDRHASGRGYRVSPEQARGGRRPSRWRDEAEDGEDWTRNGRPGLRRRSASQEDPLAGGG
MSTRG.34604.1    ------------------------------------------------------------
                                                                             


                        910       920       930       940       950       960
                 =========+=========+=========+=========+=========+=========+
Ec-14_003580     GDRYGSQAATGGGHNGEEYSRPYRFHHGSRKHKASPQSDDNNSGGYKHGGGCLPPSGKAR
MSTRG.34604.1    ------------------------------------------------------------
                                                                             


                        970       980       990      1000      1010      1020
                 =========+=========+=========+=========+=========+=========+
Ec-14_003580     WEPKKTVGAAVSPHTSRRRTVVAAGVAMGRKAAGEQSAFELTEGEAYHVRNGGGYLESLI
MSTRG.34604.1    ------------------------------------------------------------
                                                                             


                       1030      1040      1050      1060      1070      1080
                 =========+=========+=========+=========+=========+=========+
Ec-14_003580     ADKAPCNRGLNEDDGWYGGEPGRQRTPQWTENNIGRQPPGRQNSRQDNTGGPARQTGITT
MSTRG.34604.1    ------------------------------------------------------------
                                                                             


                       1090
                 =========+=======
Ec-14_003580     SVVTSRSERMPPRWIKF
MSTRG.34604.1    ---------------AP
```

```
Parameters used
Minimum Number Of Sequences For A Conserved Position: 2
Minimum Number Of Sequences For A Flanking Position: 2
Maximum Number Of Contiguous Nonconserved Positions: 8
Minimum Length Of A Block: 10
Allowed Gap Positions: None
Use Similarity Matrices: Yes
```

```
Flank positions of the 11 selected block(s)
Flanks: [34  56]  [74  109]  [128  153]  [195  206]  [222  241]  [250  267]  [326  375]  [377  433]  [452  492]  [496  521]  [555  564]  

New number of positions in Ec-14_003580.aa.fasta.trans.aa_ali.fasta-gb:  319  (29% of the original 1097 positions)
```
